# The Design of an Intelligent Robotic Wheelchair Supporting People with Special Needs, Including for Their Visual System

**DOI:** 10.3390/healthcare10010013

**Published:** 2021-12-22

**Authors:** Dorian Cojocaru, Liviu Florin Manta, Cristina Floriana Pană, Andrei Dragomir, Alexandru Marin Mariniuc, Ionel Cristian Vladu

**Affiliations:** Department of Mechatronics and Robotics, University of Craiova, RO-200440 Craiova, Romania; florin.manta@edu.ucv.ro (L.F.M.); cristina.pana@edu.ucv.ro (C.F.P.); andrei.dragomir@edu.ucv.ro (A.D.); alexandru.mariniuc@edu.ucv.ro (A.M.M.); cristian.vladu@edu.ucv.ro (I.C.V.)

**Keywords:** HMI sensors and devices, biomedical robotics, eye-tracking command system, eye gaze, assistive robotics

## Abstract

The paper aims to study the applicability and limitations of the solution resulting from a design process for an intelligent system supporting people with special needs who are not physically able to control a wheelchair using classical systems. The intelligent system uses information from smart sensors and offers a control system that replaces the use of a joystick. The necessary movements of the chair in the environment can be determined by an intelligent vision system analyzing the direction of the patient’s gaze and point of view, as well as the actions of the head. In this approach, an important task is to detect the destination target in the 3D workspace. This solution has been evaluated, outdoor and indoor, under different lighting conditions. In order to design the intelligent wheelchair, and because sometimes people with special needs also have specific problems with their optical system (e.g., strabismus, Nystagmus) the system was tested on different subjects, some of them wearing eyeglasses. During the design process of the intelligent system, all the tests involving human subjects were performed in accordance with specific rules of medical security and ethics. In this sense, the process was supervised by a company specialized in health activities that involve people with special needs. The main results and findings are as follows: validation of the proposed solution for all indoor lightning conditions; methodology to create personal profiles, used to improve the HMI efficiency and to adapt it to each subject needs; a primary evaluation and validation for the use of personal profiles in real life, indoor conditions. The conclusion is that the proposed solution can be used for persons who are not physically able to control a wheelchair using classical systems, having with minor vision deficiencies or major vision impairment affecting one of the eyes.

## 1. Introduction

The number of people with disabilities has increased significantly with the ageing of the population [1]. Out of these, people with motor disabilities are dependent on wheelchairs. Over the years, regarding the electric wheelchairs, various devices have been developed for driving the seat adapted to the level of disability the user presented [2]. Given that, statistically, more than half of wheelchair users have difficulties adapting to the user interface. The control methods were developed starting with a simple electric or pneumatic tube (blowing air into a tube) and reaching the level where the head position tracking, eyes tracking, or facial expressions are being used [3,4,5]. The insertion of mobile robotics in this field has substantially improved the control interface and of the navigation aid, factors that lead to the semi-automation of this type of locomotion. Of course, these control devices are correlated with the level of disability of the user, the automation of the user control interface is leading implicitly to the increase of the complexity of the system and the costs. However, eye-tracking devices and facial expression recognition systems are still in development, with current models offering just a few practical facilities compared to the theoretical studies [5,6,7,8,9,10,11,12]. For example, Timofei I. et al. worked on a robotic wheelchair designed for patients with severe disorders of the musculoskeletal system and other body functions. The main idea of the described project is based on the concept of “extended BCI” (brain controlled interface). The joystick control was replaced by a control unit that has an interface with an onboard computer. The BCI allows obtaining user thought-images and the power of the said thoughts, thus allowing the wheelchair to be driven using one’s “mind” [5]. In addition, Bin Hua et al. proposed a method in which there are used types of sensor data to train neural networks used to control a wheelchair robot. They used the data from a laser range finder (LRF) and a camera sensor to input the neural network. The robot is first trained by a skilful user who controls the wheelchair with the joystick in various environments. The above-said sensors collect the data that is used later to train the neural network [6]. Sudipta Chatterjee et al. propose a low-cost control method in which there are used several modules are implemented using a Raspberry Pi 4. The system uses Open CV and pre-trained models to detect more than 80 objects within an image or video stream. A separate Arduino board is used in connection with the Raspberry Pi to control the motor drive. The Arduino board also allows the control of the wheelchair using a mobile phone app. The reasoning behind this method is based on the fact that the caregivers of the elderly patients may be put in a situation in which they have to leave the house or the premises where the elderly live. In such a situation, the caregiver can extend his help to the patients by controlling the wheelchair from a distance via their mobile phone [7].

The eye-tracking devices and facial expression recognition systems are still in development, with current models offering just a few practical facilities compared to the theoretical studies [8,9,10,11,12,13,14,15,16,17,18,19,20,21,22,23,24]. In [16], Lee Y et al. propose an extended method to determine the measurement of saccadic eye movement using an eye-tracking module in a virtual reality head-mounted display. Sipatchin et al. present a case study to test empirically and discuss the hardware capabilities and visual tracking limitations of a standard VR headset with built-in eye tracking for home use in ophthalmic applications [17].

The authors have developed such a system, designed for people with severe motor impairments, which has an intelligent interface based on a combination of command methods, namely: tracking the eye gaze, the position of the head, and facial expressions. This wheelchair can be driven by direct control (successive and repetitive movement commands), semi-autonomously (repeatedly indicating visible, nearby destinations), or autonomously (indicating the destination on known routes). The experimental results presented confirm both the validity of the method and the necessity of mixing the three driving methods to eliminate the limitations imposed by using a single driving method at a given time [5,6,7,8].

The cases of major motor disabilities are caused by the alteration of the nervous system, therefore, a great number of people suffering such disabilities have strabismus-type vision deficiencies. For a correct mapping of the destination, the eye tracking system requires the tracking of both eyes. Thus, for these persons, there must be applied corrections when reading their eye gaze, but only after undergoing procedures of calibration and determination specific to each person.

The alteration of the nervous system causes the cases of significant motor disabilities; therefore, many people suffering from such disabilities have strabismus or Nystagmus type vision deficiencies. In [18], Tsai, C et al. developed a novel, fast and effective system and combined it with a low-cost eye-tracking device to quantitatively measure optokinetic Nystagmus (OKN) eye movement. In addition, Syahbana et al. designed a pupil detection method using a pattern matching approach that approximates the pupil using a Mexican hat-type ellipse pattern [19].

Our team aims to design and implement a smart wheelchair that supports people with special needs related to the control of their hands and feet. This paper deals with the problems that arise when the user of such a system has additional and important problems with his vision system. In order to obtain an intelligent control that correctly identifies the target to be reached when moving the wheelchair, these problems must be identified (measured) individually for each user.

The aim of our study is to create a smart system adaptable to most electric wheelchairs available, with an easy-to-use control interface and a semi-autonomous navigation system. The user commands the movement of the wheelchair by using the eye-tracking system, and the navigation is semi-autonomous–the user can select the desired direction by choosing one of the four commands on display: forward/backward/left/right. It is known that people who cannot move but have reasonable control over eye movements are the best candidates for eye-tracking systems. Reasonable eye movement control can facilitate follow-up, in contrast to people with different eye conditions (e.g., strabismus) or involuntary/voluntary uncontrolled eye movements (Nystagmus), which are much more difficult to track because of their movements. For a correct mapping of the destination, the eye-tracking system requires the tracking of both eyes. Thus, for these persons, there must be applied corrections when reading their eye gaze, but only after undergoing procedures of calibration and determination specific to each person.

## 2. Materials and Methods

### 2.1. An Idea of Control System

To design an intelligent mobile platform, accurate solutions to solve issues are required. The first step was to develop a real-time model, appropriately and accurately, given the environment specificities where the ensemble evolves. Next, a high-performance hardware structure capable of accomplishing the good mobile tracking trajectory, from Point A to Point B, smoothly, without any shocks, avoiding collisions and ensuring movement stability was implemented. In the third step, there was a suitable set of algorithms implemented into a software system. This system is, therefore, able to provide two functions. First, it assures the control of the actuation elements based on the information received from the sensory system. Second, the control function is based on the fact that the hardware equipment of the wheelchair provides the movement parameters according to the conditions stated above and the traffic management from intersections. On the other hand, the software system delivers a friendly user interface for the mobile system user.

Within the structure of the mobile platform, the usage of a sensory system of distance measurement is a must, as obstacles should be avoided.

To recognize the gesture of the users with special needs, we looked for a gesture tracking system. Further on, we shall isolate and analyze existing solutions proposed by existing specialized literature and the current commercial solutions in the area of human-machine interface devices using human gesture tracking such as head movement, mimicry, and eyes movement. The eye-tracking system represents a dedicated human-machine interface system used to control different elements based on human sight as the source of the input data. The head tracking system is involved in the hands-free control of some IT devices as an essential aspect for individuals with a relatively high degree of physical disability—especially in the moving area.

After obtaining informatics, electronics, and economic insight into the field and the solutions delivered by specialized literature and the commercial area, the lab research consisted of modelling and simulations.

This process resulted in the current architecture of the system and is composed of three main subsystems. First, the movement system has two subcomponents: the electric wheelchair, whose motor system has been adapted for automatic control and the sensory system that takes over and processes data related to the travel environment (3D environmental mapping system and proximity system). The command-and-control system is the second principal component of the system. These are two subcomponents: the sensory system for taking orders from the user (tracking eye movements, head movements and facial expression) and the control system that runs the algorithms needed to establish the destination point in the 3D environment and establish the trajectory this point.

### 2.2. Sensorial System Architecture

Given the analysis of the previously obtained solutions as part of the existing specialized literature, various mobile platforms using two or three scanning sensors, such as a LiDAR sensor, capable of creating three-dimensional maps of the environment of the mobile platform, have been identified. Given the high price of such LiDAR sensors, we propose an option where only one 3D LiDAR sensor could be used. This main LiDAR [25] sensor should be able to provide three-dimensional mapping of the environment where the mobile platform is moving, and at the same time to gather and supply information on the existing obstacles, with compact dimensions, ensuring the necessary measurement accuracy and being safe for the human eye. Considering the configuration of the mobile platform, the use of a single LiDAR 3D device generates several “blind spots” in its immediate vicinity. Therefore, other 2D LiDAR [26,27] sensors were used as auxiliary sensors with a considerably lower price. This way, the system will offer a global image of the environment where the mobile platform is moving. The sensor system required for 3D mapping has been completed with a proximity sensors system to cover its “dead zones” and avoid accidental collisions with dynamic objects with high movement speed.

The commands and messages were interfaced on display, a component of the system. In addition, a 3D mapping system of the environment permanently provides topometry data to the design and a visual image on display (based on system commands or messages).

We used three methods to control movement. By tracking head movements for direct control of primary actions of the wheelchair (forward, backward, rotation, etc.). Second, we are tracking eye movements for the destination point set, located nearby, in a visible area. This method allows short, successive trips to reach the final destination. Third, tracking eye movement to stabilize the goal in a partially visible environment, with known or unknown topography.

For the direct control, we used the tracking of the head movements for simple rules of action, rotation, stopping, and the correlation of the activities with the amplitude and speed of motion of the head. In addition, certain gestures (behavioural patterns) were correlated with emergency actions (for example, the sudden lowering of the head with the emergency braking during the collision) [23]. Semi-autonomous driving was achieved by tracking the direction of the gaze with a sensorial system (eye gaze, head movements, facial expressions). The sensory system also interfaces with a VHF + navigation system to specify the destination in a known (or visible) space [24].

The facial expression tracking was used to confirm the person’s ability to perform the control, detecting primary expressions such as satisfaction, surprise, horror, boredom, etc. Then, depending on the facial expression, the movement was blocked, continued, or assisted by messages needing confirmation.

### 2.3. Design of the Wheelchair

The basic structure consists of an electric wheelchair on which the systems mentioned above were mounted. A tablet provides the visual interface between the system and the user. The tablet allows the visualization of the 3D environment in which the wheelchair moves both by specifying the destination point entered in the map, and by marking the evolution on the chosen trajectory. It can also use this interface to control primary movements through the eye gaze.

The sensory system for 3D mapping of the environment is mounted on the chair’s back, ensuring optimal visibility. The proximity sensor system is mounted on the sides of the seat, covers both the dead areas from the first system, and avoids obstacles with high-speed mobility.

**Figure 1:**
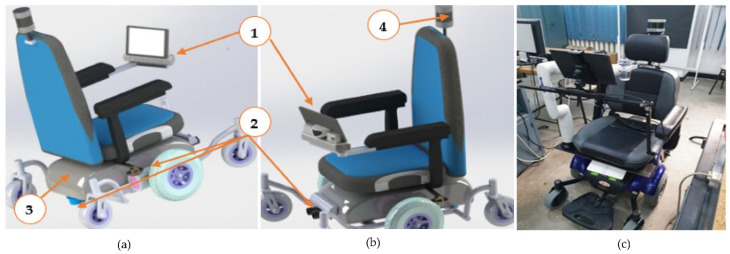
Intelligent wheelchair (**a**) rear-view, (**b**) front-view; 1—sensorial system (eye gaze, head movements, facial expressions); 2—proximity sensorial system; 3—energetic system; 4—3D mapping system; (**c**) the current implementation of the sensory system on the autonomous wheelchair.

Two schematics and one real image of the intelligent wheelchair are displayed in Figure 1.

The sensory system for tracking the movement of the head, following the eye gaze and facial expression, is mounted on the side handles of the chair through a mobile mechanical system that allows users easy access to sit in the chair or leave it. Under the effective seat is the electronic system and control system, as well as the energy system of the wheelchair.

Figure 1c shows the current implementation of the sensory system on the autonomous wheelchair. This intelligent wheelchair solution was the result of the project “Designing, modelling and simulation in the operation of distributed configurations of sensors and visual serving systems on autonomous systems complex (SAC-SI, SAC-ARP, SAC-VAM) for medical personnel- social assistance technologies, intra/extra hospital and home”, which was part of the complex research project: “Intelligent and Distributed Management of Three Autonomous Complex Systems Integrated into Emerging Technologies for Personal Medical Assistance and Service of Flexible Precision Manufacturing Lines (CIDSACTEH)” [28].

### 2.4. Ethical Considerations

All the measurements described in this section were designed and conducted considering the Ethical Principles for Medical Research Involving Human Subjects—Declaration of Helsinki developed by The World Medical Association (WMA), and all the applicable national legislation–Law 43/2003 regarding the patient rights, Law 95/2006 regarding the health care reform, Ministry of Health Order No. 1502/2016, national guidelines for ethical principles in medical research on human subjects. All the participants were informed about the purpose of the measurements study, the goals it proposes, the expected duration, the procedures that will take place, the known risks and the inconveniences that it may cause, the expected benefits, and how the data gathered during the measurement study is anonymized, and may use it for research activities, education, the scientific research publication of the research results. All the participations were made voluntarily, without being paid. All the participants were willing to participate signed Consent forms, which detailed all the information mentioned above. Since all the measurements were conducted using non-invasive, contactless devices and no administration of medical drugs were required in the process; there was no requirement from the Ethical Commission for research involving human subjects. To assure the anonymization of data, each subject’s dataset was coded using capital letters, i.e., Subject (A), Subject (B), and so on.

To assure that all the ethical principles were respected during our research work, we contracted an external consultant, RehabMed, which assisted us during all the research phases.

### 2.5. Methods

People with special needs related to controlling their hands and feet may have additional and essential problems with their vision system. The paper presents the methods used to determine visual deviations and compensate for errors induced in the driving system.

Given the TRL development of our work (Level 2/3), initially, the measurements were designed on a two-stage architecture: on the first stage, we were looking for the limitations of the sensory system; on the second stage, we intended to conduct measurements for healthy subjects, in laboratory conditions, to create individual customer profiles for the human–machine interface. However, while performing these measurements in public areas of our faculty, a person with mobility and vision pathology (Subject H) requested us to be involved as a subject in our research. Thus, we reorganized the measurements in a three-stage architecture:

- In the first stage, we were looking for the limitations of the sensory system.
- In the second stage, we conducted measurements for healthy and vision-impaired subjects in laboratory conditions to create individual customer profiles for the human-machine interface.
- In the third stage, we conducted measurements to evaluate the human–machine interface in real indoor conditions for a vision and mobility impaired subject.

Two different methodologies were used to record the measurements presented on the current paper.

#### 2.5.1. Methodology A

This method was designed for the measurements conducted to validate the sensory system and determine its use limits. This measurement methodology, for a specific environmental (indoor lighting) condition, is adapted after [12]:

- The research team checks the sensing device in respect to the display;
- One team member notes the measurement conditions, subject and location where measurements are saved;
- Another team member explains the measurement protocol to the subject;
- A third member operates the measurement software (checks if the system is loaded with the subject profile, or creates a new profile if the subject is presented for the first time to the sensing system; executes the measurement protocol; etc.)
- The team conduct a measurement simulation to accommodate the subject with the measurement and to validate that de subject understood the measurement protocol;
- Conduct the measurement protocol;
- Allow the subject to rest; verify the integrity and the validity of the recorded data;
- Repeat the last two steps five times, each time for a different target point (as described on the following measurement protocol) and for a proper measurement protocol.

The result is a measurement set of five recordings, one for each target point.

To determine the existence of static errors, a measurement set consisted of recording the sensed gaze location for five target points. Four target points were located in the corners of the image and one in the centre of the image. The target point locations were expressed in pixels for an HD image (1920 × 1080 pixels); the corner target points were placed 100 pixels away from the image margin (horizontal and vertical).

The measurement protocol consisted of the following steps:

- Display the target point as white over a warm colour background for 3 s to allow the user to locate it; no data are recorded;
- Display the target point as white over a black background for 5 s, gaze related data are recorded at a sampling rate of 80 values per second;
- The target points are displayed in the following order: upper left, upper right, centre, lower left, lower right.

**Figure 2:**
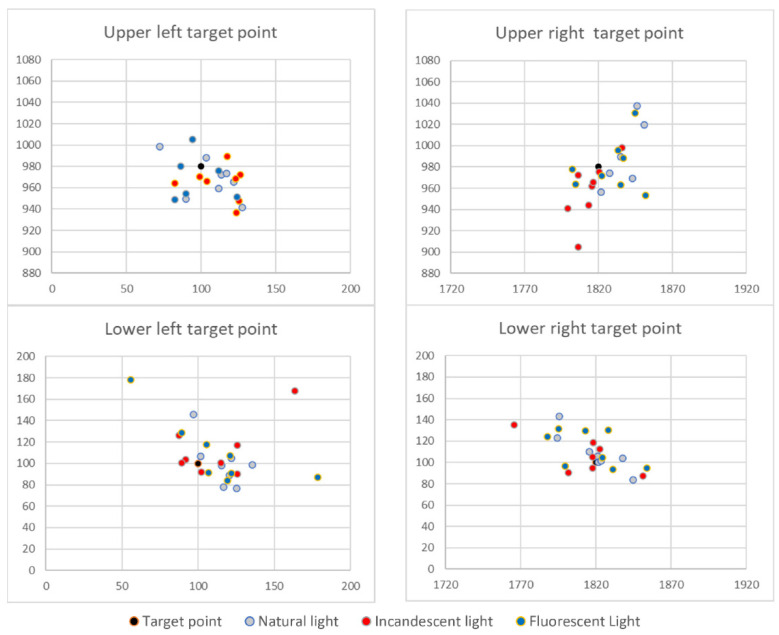
Centroids dispersion for corner target points in different lighting conditions.

**Figure 3:**
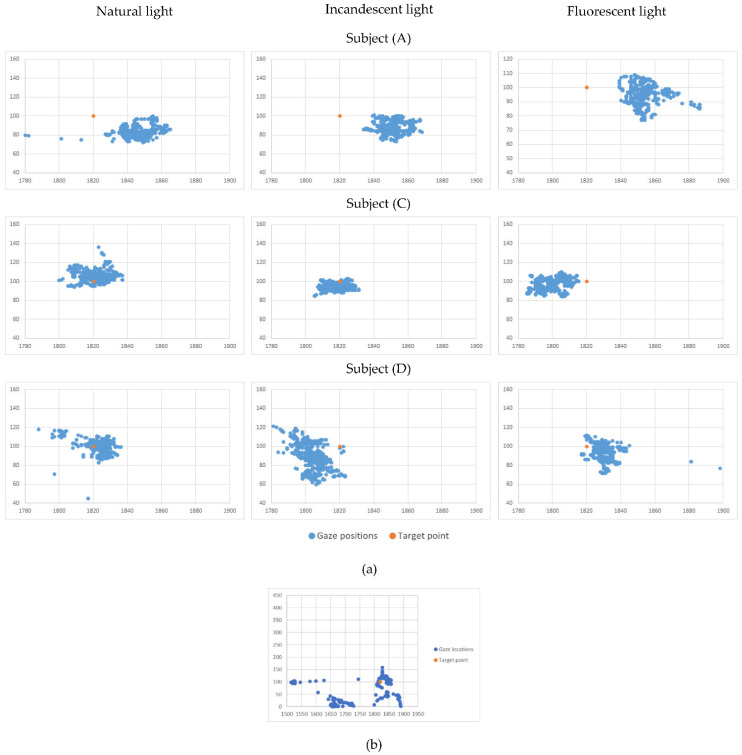
(**a**) An example of recorded gaze dispersions per subject and environment conditions for the lower right target point; (**b**) Subject (H) gaze positions represented on a scale which to allow the representation of all gaze locations.

**Figure 4:**
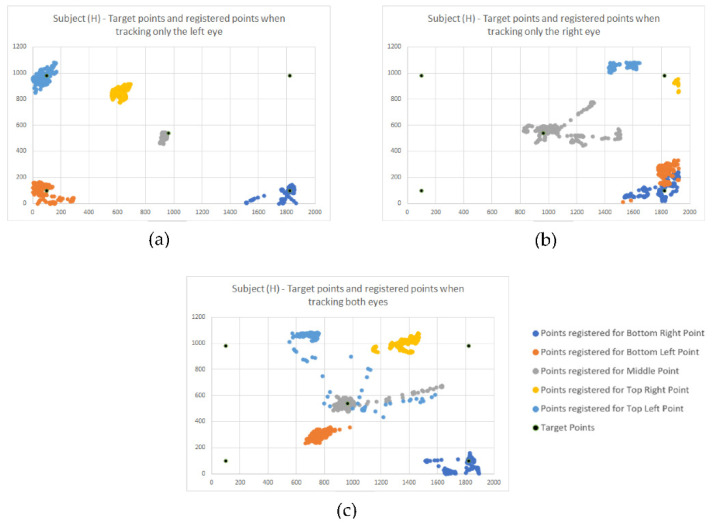
(**a**) Plot of gaze dispersions recorded for the right eye, all target points, (**b**) plot of gaze dispersions recorded for the left eye, all target points, (**c**) plot of gaze dispersions recorded for both eyes, all target points.

For each measurement set, we computed the following information:

- The range of dispersion to determine the outliers of gaze for each target point (Figure 2, Figure 3 and Figure 4);
- The variance and standard deviation of gaze for each target point;
- The centroid of data points (recorded gaze positions), and;
- The accuracy of gaze concerning the target point.

#### 2.5.2. Methodology B

This method was designed to obtain immediate feedback about the proposed solution in a real-life scenario. In addition, the research team considered that it is essential how a beneficiary perceives the proposed system. Thus, Subject (H) offered to evaluate the proposed solution by driving the smart wheelchair on the hallways. In this sense, the research team elaborated the following measurement methodology:

- The research team checks the sensing device in respect to the display;
- The research team checks the intelligent wheelchair systems, that they are working correctly;
- The research team checks that the safety measurements are active and functional;
- A team member notes the measurement conditions, subject and location where measurements are conducted, and where the recorded data are saved;
- A team member explains the measurement protocol to the subject;
- A team member explains how the intelligent wheelchair can be controlled, details the options displayed on the HMI, and how the subject can generate commands; also, explains the safety protocol, in case of unexpected behaviour of the intelligent wheelchair;
- A team member verifies the measurement software (checks if the system is active and loaded with the subject profile; executes the measurement protocol; etc.)
- The team conduct a measurement simulation to accommodate the subject with the measurement and to validate that de subject understood the measurement protocol;
- Conduct the measurement protocol while the subject manoeuvres the smart wheelchair; The manoeuvring time is at the subject’s discretion;
- The subject can opt to take breaks at any point during the test; while on break, a team member verifies the integrity and validity of the recorded data.
- When the subject decides to end the test, a team member verify the integrity and the validity of the recorded data.

The measurement protocol consisted in:

- Display the subject customized HMI;
- User selects at his free will what commands to give to the smart wheelchair
- The measurement software records in the background:
  - Raw gaze position on the HMI;
  - The sensing system state: user detected or not detected;
  - The sensing system state: gaze detected or not detected;
  - When the subject looks toward the command buttons displayed by the HMI.

### 2.6. Measurements

The sensing device datasheet stated that the product was designed to be operated on indoor conditions, given its constructive water ingress protection. Nonetheless, we tested the device in non-raining outside conditions. We published the results in a previous paper–the conclusion was that the sensing device could be used outdoors only if there is a cloud ceiling since direct sunlight can “blind” it. Therefore, now, we focused on testing the device in indoor conditions. In real-life scenarios, indoor illumination may be assured by natural light, warm light (incandescent bulbs or warm light LEDs) or cold light (fluorescent or cold light LEDs). Thus, the measurement protocol was conducted for each one of these three lighting conditions.

#### 2.6.1. The First Stage of Measurements—Sensory System Validation

We conducted a series of measurements on healthy subjects to validate the sensory system and determine its use limits. The measurements were designed to evaluate the influence of environmental factors, the static errors related to eye gaze, the possible effect of eyeglasses over the measurements, and the possible impact of the subject’s age. In this stage, the measurements were conducted on a set of healthy subjects, among which some were wearing eyeglasses—for these subjects, the measurements were performed both with and without eyeglasses. The methodology used for these measurements was Methodology A. An example of recorded data, for one subject, in different lighting conditions is illustrated in Figure 2.

#### 2.6.2. The Second Stage of Measurements—Personal Profiling

This stage in our measurement architecture aimed to explore the possibility and the modality of creating individual-tailored user profiles for the human-machine interface. The methodology used for these measurements was Methodology A. We started by analyzing the recorded measurement dispersions for each subject and each target point—a volume of more than 200 datasets. In this phase, we used two types of instruments: plots representing close-ups for the group of points (representing recorded gaze positions), as shown in Figure 3, which allowed us to have an image about the point dispersion properties; the other type on the instrument was the gaze statistic records as shown in Table 1 and discussed in the Results section of the paper.

The following measurements were conducted on Subject (H) (strabismus and nystagmus pathology) and two randomly chosen subjects from (A) to (F) (healthy subjects), namely Subject (C) and Subject (F), forming a control group. We considered this number of subjects satisfactory in correlation with our TRL development (at this stage, reaching Level 3). Further development will consider subjects with different degrees of Nystagmus and with varying types of strabismus.

For the first challenge, we used one of the advanced modes offered by the sensing device, which allows us to track both eyes gaze and individual gaze for each eye. Using the simplified version of measurement methodology and protocol, as described in Section 3, now we conducted a series of measurements tracking both eyes, then just the left eye, then the right eye. The results obtained for Subject (H) are presented in Figure 4a–c.

#### 2.6.3. The Third Stage of Measurements—Primary Evaluation of the Human-Machine Interface in Real-Life Indoor Conditions

The last stage of our measurements was to obtain immediate feedback about the proposed solution in a real-life scenario. Firstly, the team members validated the HMI by testing the intelligent wheelchair movement under eye gaze command on the faculty hallways. Among the advanced functions provided by the sensing system is the “user detected or not detected” function. This function signals that a subject is present in the sensing system field of view, but attention is not directed to the HMI but other events or elements in the environment. This signal is used by the intelligent wheelchair safety software to know when the user is distracted. However, this subject is not relevant to the current research work. In addition, another function signals if the gaze is detected or not, and it is also an input for the safety software. In parallel with the feedback offered by the subject, the purpose of those measurements is to evaluate the amplitude of the “gaze lost” phenomena, as detected during the personal profiling stage. The methodology used for these measurements was Methodology B.

## 3. Results

In this section, we presented some results obtained from the tests performed on the participating subjects, namely: Table 1 showed the statistical gaze records of Subject F; Table 2 shows personal profiling for Subject H whit the statistical gaze records; Table 3 shows the percentage of points in a radius of 100 pixels around the target point for three of the Subjects H, C and F.

**Figure 5:**
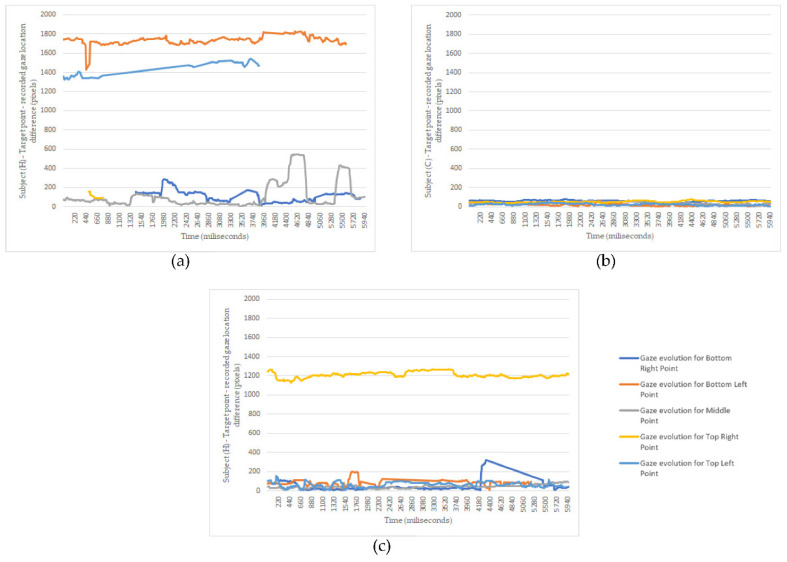
(**a**) The evolution in time of the difference between the target point and recorded gaze location, as determined for Subject (H). The gaze was considered for the right eye, (**b**) the evolution in time of the difference between the target point and recorded gaze location, as determined for Subject (C). The gaze was considered for the right eye, (**c**) the evolution in time of the difference between the target point and recorded gaze location, as determined for Subject (H). The gaze was considered for the left eye.

Figure 5 displays the absolute differences, computed in pixels, between the target point locations and the right eye gaze locations, as an evolution in time. According to individual recordings for each of the 5 target points, there are five different evolutions, but we have chosen to display them superposed on a single plot. The zero moments on the time axis corresponds with the start of the recording. One can observe that some recordings are missing—i.e., gaze evolution recorded when targeting the top left target point, which is missing after 4 s. This is because eighter the gaze is directed toward somewhere outside the display margins, the sensing device entirely loses eighter the gaze.

## 4. Discussion

Electric wheelchairs were controlled with a joystick, but people with musculoskeletal diseases/injuries resulting from various reasons, be they accidents or genetic inheritance, cannot use the joystick for manual control. Starting from this problem, the use of the control of the different dispositions with the help of eye-tracking was an optimal solution and gave results. Eye-tracking is a process of estimating a user’s gaze or where the user is looking. Several studies and articles have been written on eye-tracking, which present current developments, comparing different estimation techniques, configurations, and applications to the input size. For example, ref. [29] shows a review of eye gaze tracking technologies with improved usability that allow free head motion, excluding the need for a calibration procedure and also describes in detail how the pupil-corneal reflection technique is not yet an ideal solution for general interactive applications. The researchers in [30] describe the challenges in developing an available video-based eye detection and tracking technique due to numerous factors such as different light conditions or individuality of eyes and compares current methods for gaze estimation considering their geometric properties and accuracies. In addition, various metrics in which eye movements can be systematically measured and their advantages have been studied to verify the usability of a human-machine interface, as it turns out [31]. In [32], a new gaze detection method uses adaptive boosting and continuously adaptive mean shift methods to generate inputs for a support vector machine-based classifier to detect the eye region and secondly applies edge detection and binarization methods to calculate the centre of the pupil. Lee K.F. et al. developed a head-mounted device to track the eye gaze and estimate the gaze point on the user’s visual plane to be used in assistive devices and human-computer applications. The conducted experiments proved that the device is very efficient in terms of performance and cost-effective due to all the components used as part of the device [33]. In [34], eye tracking devices were used to develop an application that can help architects and designers to make accessible environments for people with motor disabilities that can have problems identifying and selecting the correct route when navigating inside buildings. Michael A. E et al. conducted a series of experiments involving an eye-controlled wheelchair in which twelve amyotrophic lateral sclerosis (ALS) patients participated. The patients had to perform different tasks in controlling the wheelchair using their eye gaze. The results were remarkable, proving that an eye-controlled wheelchair can be an optimal solution for ALS patients and other groups with motor impairments [35].

Initially, these eye-tracking techniques were used in neurology, ophthalmology and psychology to study various forms of oculomotor nerve manifestation. Saccadic eye movement can help researchers establish the relationship between cognitive function and psychiatric disorders. Eye-tracking devices have been used to measure the saccadic eye movement system. For further information, an overview of saccadic eye movements can be found at [36]. Then they were used in human interaction with smart devices (computer, tablet, smartphone), virtual reality, advertising design, diagnosis of certain diseases, and the study of human behaviour. Magee J.J. et al. developed a communication interface for people with severe cases of paralysis called EyeKeys. The interface takes input from a USB camera, and applying unique algorithms can detect the user’s eye gaze.

A video game was developed to evaluate the performance of EyeKeys by comparing it with the standard mouse input. This experiment proved that such a system could be a practical solution for people with severe paralysis [37]. In their research paper, S. S. Liu et al. compare camera-based and infrared sensor/emitter eye tracking systems and how the latter has more advantages when considering the cost, user interaction, and eye strain reduction [38]. In [39], how electronic consumers perceive images online was investigated. The eye-tracking experiment was performed on several 33 participating women looking at 74 images randomly displayed with handbags. In addition to providing concrete evidence of the visual attention of the female participants, the study gives images with bags for women. It also expands the existing knowledge about the observable behaviours observed in electronic consumers. The evidence obtained can be further applied in empirical research and establish a visual behaviour theory for consumers. Although they were not included in this study, elements of colour, material, etc., factors that influence observable behaviour and the cognitive process of electronic consumers has not been discussed. The paper [40] presents another possibility of applying eye-tracking technology and examines the possible impact of visual marketing incentives on Travel 2.0 websites (T2W) application user behaviour. The results showed a higher advertising efficiency in the case of the hotel’s social network. However, limitations were also observed due to the sample size and sampling method, the specific choice of T2W and mode navigation of participants. In [41], it was wanted to test the combination of Neurocode-Tracking with commercially available eye-tracking. Ten male subjects were exposed to a reference recording condition (watching a fixed cross on the screen), followed by five TV commercials representing five different banks. Experiments have shown that this combination can be used successfully in advertising research and provides valid information on individual and group cognitive dependence and emotional responses that lead to objective evaluation of TV commercials.

In [42], an eye-controlled wheelchair was proposed using an infrared camera equipped with LEDs for automatic dimming. It uses a gaze estimate using the pupil’s knowledge: size, colour, shape, pupil location detection and then converting the pupil location based on a simple eye pattern it becomes a wheelchair control for a certain threshold angle. Although the processing time of the algorithm was fast, it has some limitations, so the threshold used was only suitable for a particular lighting condition, degrading significantly when intense lighting occurs (e.g., sunlight). Another impediment in the control of the seat was that it stops when the user blinks and when the gaze deviates from the direction key, and for moving forward, the user must look down, which is impractical. Therefore, it did not prove a good performance in avoiding obstacles, and the algorithm can be used only to avoid tiny obstacles. In [43], for example, the image of the eye was divided into nine blocks of three columns and three rows. Based on the location of the pupil centre, the output of the algorithm was an electrical signal that controlled the direction of movement (left, right, forward) of the electric wheelchair. However, this study did not assess response parameters, such as speed and accuracy as changes in lighting conditions, nor did the safety parameters for the wheelchair be analyzed in the event of obstacle detection. The eye controls another type of wheelchair by processing images of a head-mounted camera using Gaussian filtering to remove Gaussian noise from the image [44]. The wheelchair moves in three directions (left, right and forward) based on the relative position of the iris, and the start or stop is made by blinking for 2 s. However, this study did not analyze the system’s performance during the transition of pupils from one direction to another.

The optical eye-tracking system that controls an electric wheelchair by translating the user’s eye movement to specific points on a screen has been used by several researchers.

All these studies have been performed in case the eyes are functionally healthy. However, what happens when the wheelchair user also has visual impairments, such as strabismus and Nystagmus? Very little research has been reported on the technique of eye-tracking for the diagnosis of strabismus and Nystagmus. For example, in [45], a Tobii device was used as the eye-tracking tool to obtain eye-characteristic data on an ophthalmic examination (including strabismus), calculating the deviation of the eye data. However, this method was proposed at the prototype level only, without presenting accurate data on the strabismus look to demonstrate the prototype’s performance. Another approach [46] is based on eye-tracking for the Hirschberg test. Although it is a classic method of measuring binocular ocular misalignment, the performance of their system was studied only on five healthy subjects, and the effectiveness of the method for determining strabismus was not tested. Finally, in [47], a tool for measuring gaze direction to estimate the angle of strabismus was presented. Still, due to the relatively small number of participating subjects (only three subjects), it is not possible to prove the effectiveness of its use. The research in [48] also proposes an assisted training system for people with mild strabismus eye muscle movements. The system includes training software, a portable eye tracker (Tobii X2-60), and a laptop. The eye tracker is mounted under the laptop monitor, and a tracking target is displayed on the monitor. Although promising, experimental results were presented and the feasibility of the proposed training system was not fully demonstrated due to the small number of participants, the need to introduce a timer into the system to avoid additional training time that induces eye fatigue was highlighted.

In our study, experiments have shown that prior profiling of the system for each person is necessary to increase accuracy. Only one visually impaired subject is shown in the paper to illustrate the particular situations that can occur. However, from tests and medical information, the authors have established that the visual problems of these patients generate significant and varied challenges that cannot be generalized. Therefore, it is necessary to calibrate and adapt the system for each subject.

For patients without visual impairment, the allowed and accepted errors for the correct operation of the system were quantified.

To create an application that will be useful for people with special needs, regardless of whether they have different visual system disorders, an algorithm will be implemented to correct their deviation based on the results of the previously conducted tests.

### 4.1. Sensorial System Validation

By analyzing all the recorded data from all the subjects, the research team concluded no significant differences among environmental conditions; thus, the sensing system behaves stable and consistent. Initially, we expected to notice incandescent lighting conditions perturbations since the infrared light component might interfere with the sensory system working principle; but our assumptions were infirmed. A more powerful IR source, such as direct exposure to the sunlight, is needed to interfere with the sensory system. In addition, there are no elements which to indicate the existence of static errors in the sensing system.

By analyzing the positions of the centroids about the target points, we concluded that, in relation with the display used in this stage (resolution of 1920 × 1080; we considered that conversion to metric or imperial measurement units is irrelevant at this stage since all the information is processed in pixel measurement units), the command buttons shown on the displayed interface should have a minimum dimension of 100 pixels, with a minimum separation between two command buttons of 50 pixels (Figure 5), to assure an interface viable for all subjects. This led us to investigate individual patterns for each subject; the measurements conducted are presented in Section 2—the second stage of measurements—personal profiling.

### 4.2. Personal Profiling

The measurements presented in the *Second stage of measurements–Personal profiling* revealed that each subject exhibits different gaze behaviour. Figure 3 illustrates close-ups for the group of points (representing recorded gaze positions around the lower right target point) for three randomly chosen subjects in different lighting conditions. Subject (A) exhibits good precision but low accuracy. Subject (C) exhibits perfect precision and good accuracy. At the same time, Subject (D) indicates a low precision and a medium accuracy. Those conclusions were consistent no matter the lighting conditions, as already concluded in the previous section. This made us furthermore conclude that we can improve the human–machine interface by creating individual profiles based on the subject’s specifics: for Subject (A), we can perform a correction of the sensing data to improve the accuracy to increase the success rate of given commands on the human–machine interface (HMI); for Subject (C) we can increase the density of command buttons displayed on the (HMI) since the subject can easily gaze-target small areas on the HMI image—thus we can display a more significant number of possible user commands; in reverse, for Subject (D), the HMI must show larger command buttons, to increase the success rate of given commands–since the Subject (D) seems to be prone to scatter the gaze over a larger area. A brief reminder: the information displayed in Figure 3 represent recordings for the gaze of healthy subjects.

In the next phase, motivated by the desire to create an application that will be useful for people with special needs, regardless of whether they have other disorders of the visual system, we have conducted, on the subject of mobility and vision pathology, a simplified version of the Methodology A (disregarding the lightning conditions, since they do not interfere). First, we analyzed the close-ups for the group of points (representing recorded gaze positions around the target points). As a result, we found a way larger dispersion of the points—Figure 3b illustrates the dispersion on a scale that allowed us to display the entire dataset of recorded gaze locations—to be noted that the 1:1 ratio between axes was maintained, to assure the correct geometric representation.

Subject (H) is officially diagnosticated with two vision disorders: strabismus and Nystagmus. The subject’s condition raised two challenges for our research work: determining the subject’s gaze intention toward a specific command displayed on the HMI (induced by the strabismus) and how to tackle the uncertainty with the highest precision and accuracy caused by the Nystagmus.

Figure 4a displays the gaze dispersions recorded by tracking the right eye only. One can observe that the recorded gaze datasets overlap, even if the intended target points are different. Thus, the information obtained by tracking only the right eye cannot be used as input for the HMI.

Figure 4b displays the gaze dispersions recorded by tracking the right eye only. It shows good accuracy and good precision, except the precision determined for the top right target point. Further measurements will help us understand this situation.

Figure 4c displays the gaze dispersions recorded by tracking both eyes. Excepting the centre point and the lower right point, which were recorded fair datasets, the information obtained by monitoring both eyes cannot be used as input for the HMI.

These findings are also sustained according to statistic data, which is represented in Table 2.

By analyzing this data along with gaze dispersions shown in Figure 4a–c, we concluded that Subject (H) dominant eye is the left eye, and tracking the gaze only for this eye will significantly improve the interaction with the HMI, thus resulting in another individual-tailored user profile.

Since this data shows low-quality values for accuracy, we explored the analysis towards the percentage of points registered in the vicinity of the target point, defined as a circle with a radius of 100 pixels.

The determined data for the Subject (H) versus the data determined for the control group is reproduced in Table 3.

All this statistic data presents plots of recordings, but it does not consider the time evolution. Thus, next, we focused on analyzing how the recorded gaze locations evolve in time.

As for the discussion, Figure 5 confirms our initial suppositions. The subject’s left eye (H) is the dominant one, and its gaze can be used as command input since the deviations around the target points are in a vicinity of fewer than 200 pixels (Figure 5c). The variations oscillations are consistent with a small nystagmus effect. The top right error tracking, shown in Figure 4b, is observed here too. As for the right eye of Subject (H), the Nystagmus induces robust gaze oscillations, thus making it improper to be used as the input signal. In addition, Figure 5c reveals the possibility of using a filtering method to increase the accuracy of gaze as the input signal. Another vital piece of information indicated by those graphs is the moments when the gaze is lost. This determined us to record additional information during the third stage of measurements.

The research team gained experience measuring and evaluating data to create personal profiles for future subjects by completing this measurement stage. These personal profiles are used to improve the HMI efficiency and to adapt it to each subject needs.

### 4.3. Primary Evaluation of the Human-Machine Interface in Real-Life Indoor Conditions

The subject used the smart wheelchair for a total time of 15 min. For this time, the following data were acquired:

- The percentage of time while the system detected the subject. The most extended period of the user not being noticed was 2 s.
- The percentage of time while the system detected the subject’s gaze. The most extended period for “gaze lost” was 2 s, but the “gaze lost” event occurred 39 times over the 15 min of intelligent wheelchair operation. The most extended period of uninterrupted “gaze detected” was 111 s.
- However, the user was able to provide valid commands only for a small fraction of time. This is due either to the difficulty to target the command buttons, or due to the anxiety of using the system. This finding requires more in-depth research, measurements, and analysis, which will be performed as future work.

### 4.4. Limitations of the Our Study

From the experiments performed in our study, for visually impaired people, the profiling process determines the error given by the vision impairment, which leads to case determination:

- Vision deficiencies within minor error limits—both eyes are followed, additional correction is performed via user profiling, or further confirmation of the given commands is required (case of Subject (A) and Subject (D)).
- Major vision impairment in one eye—the eye to be tracked (the dominant, less impaired eye) is determined, and only step-by-step movements can be made using the primary movements.
- Major visual impairment—the system cannot be used.

## 5. Conclusions

The main application has the scope to design an intelligent wheelchair HMI, able to navigate towards a 3D destination in the working space. The user of the supporting mobile robot can watch a 2D image corresponding to the 3D reality around this position. The control system identifies the 2D point where the user’s eyes are focused and find the corresponding 3D point from the real world. To use the intelligent wheelchair, each user with special needs must accomplish a calibration procedure. This procedure implies several measurements regarding the properties/problems of the patient visual system. For users having no problems with their optical system, the main task of the control system remains to differentiate, based on the results of the calibration procedure, between different users. By using the information from the intelligent sensor system, the wheelchair will travel towards the destination, avoiding collisions. For users with problems regarding their visual systems (strabismus and Nystagmus), the control systems must be upgraded with unique navigation algorithms.

## Figures and Tables

**Table 1:** Example of a gaze statistic records for a Subject F.

Gaze Statistics Subject (F)										
		Range (Interval in Pixels)		Variance (Squared Pixel Value)		Standard Deviation (Pixel Values)		Centroid (Coordinates in Sub-Pixel Precision Values)		Accuracy (Sub-Pixel Precision Values)
		X	Y	X	Y	X	Y	X	Y	
Point 1	Natural light (250 lux)	29	21	38.04	20.09	6.16	4.48	117.05	973.62	18.21
	Fluorescent light (700 lux)	19	25	15.42	33.29	3.92	5.77	111.66	976.02	12.32
	Incandescent light (400 lux)	20	30	18.95	42.85	4.35	6.54	123.02	968.20	25.87
Point 2	Natural light (250 lux)	31	31	53.41	45.49	7.30	6.74	1835.16	989.18	17.72
	Fluorescent light (700 lux)	26	33	32.89	53.34	5.73	7.30	1836.59	988.22	18.51
	Incandescent light (400 lux)	37	24	96.66	21.78	9.83	4.66	1836.04	997.90	24.03
Point 3	Natural light (250 lux)	27	41	57.09	77.59	7.55	8.80	966.32	555.28	16.54
	Fluorescent light (700 lux)	25	27	28.95	51.30	5.38	7.16	971.94	559.21	22.63
	Incandescent light (400 lux)	27	35	29.38	59.05	5.42	7.68	974.22	571.57	34.62
Point 4	Natural light (250 lux)	34	28	76.85	28.37	8.76	5.32	121.71	104.99	22.28
	Fluorescent light (700 lux)	18	20	16.33	17.80	4.04	4.21	120.85	107.45	22.14
	Incandescent light (400 lux)	48	24	132.55	27.91	11.51	5.28	125.73	116.90	30.79
Point 5	Natural light (250 lux)	29	24	53.41	45.15	7.30	6.72	1823.58	101.73	3.98
	Fluorescent light (700 lux)	22	16	25.71	18.11	5.07	4.25	1824.50	104.54	6.40
	Incandescent light (400 lux)	25	25	26.30	26.36	5.12	5.13	1817.97	105.34	5.71

**Table 2:** Example of a gaze statistic records for a subject H.

Gaze Statistics Subject (H)										
		Range (Interval in Pixels)		Variance (Squared Pixel Value)		Standard Deviation (Pixel Values)		Centroid (Coordinates in Sub-Pixel Precision Values)		Accuracy (Sub-Pixel Precision Values)
		X	Y	X	Y	X	Y	X	Y	
Point 1	Both eyes tracked	1034	645	34,535.49	45,301.3	185.83	212.84	784.03	956.22	684.44
	Left eye tracked	165	230	1162.82	1000.42	34.10	31.62	64.30	953.41	44.50
	Right eye tracked	217	75	5010.15	382.75	70.78	19.56	1533.26	1053.10	1435.12
Point 2	Both eyes tracked	320	151	4514.04	925.80	67.18	30.42	1347.11	1000.79	473.34
	Left eye tracked	132	141	956.37	955.06	30.92	30.90	618.54	845.45	1208.97
	Right eye tracked	32	100	138.66	585.42	11.77	24.19	1903.14	918.96	103.14
Point 3	Both eyes tracked	773	197	23,736.12	1321.39	154.06	36.35	988.02	544.99	28.46
	Left eye tracked	51	88	102.34	318.56	10.11	17.84	931.05	508.63	42.68
	Right eye tracked	680	332	22,099.76	2663.84	148.65	51.61	1035.18	565.08	79.25
Point 4	Both eyes tracked	316	121	1606.28	803.45	40.07	28.34	770.48	297.23	698.89
	Left eye tracked	284	162	4538.12	3201.23	67.36	56.57	83.93	100.05	16.06
	Right eye tracked	393	316	1313.04	2143.85	36.23	46.30	1825.88	252.24	1732.59
Point 5	Both eyes tracked	376	159	11,050.19	1918.58	105.11	43.80	1771.85	67.28	58.20
	Left eye tracked	355	141	2328.02	1174.66	48.24	34.27	1812.92	100.28	7.08
	Right eye tracked	383	221	10,502.52	4312.55	102.48	65.67	1803.55	127.96	32.44

**Table 3:** Percentage of points in a radius of 100 pixels around the target point.

Percentage of Points in a Radius of 100 Pixels around the Target Point				
		Subject (H)	Subject (C)	Subject (F)
Point 1	Both eyes tracked	0%	100%	100%
	Left eye tracked	98.95%	100%	100%
	Right eye tracked	0%	100%	100%
Point 2	Both eyes tracked	0%	100%	100%
	Left eye tracked	0%	100%	100%
	Right eye tracked	88.88%	100%	100%
Point 3	Both eyes tracked	92.43%	100%	100%
	Left eye tracked	100%	100%	100%
	Right eye tracked	73.24%	100%	100%
Point 4	Both eyes tracked	0%	100%	100%
	Left eye tracked	93.16%	100%	100%
	Right eye tracked	0%	100%	100%
Point 5	Both eyes tracked	67.76%	100%	100%
	Left eye tracked	97.41%	100%	100%
	Right eye tracked	51.16%	100%	100%
